# Huge aneurysmal bone cyst secondary to giant cell tumor of the hand phalanx: a case report and related literature

**DOI:** 10.1186/s12885-020-06746-z

**Published:** 2020-03-19

**Authors:** Mingzhuo Li, Yaokai Gan, Dingwei Shi, Jie Zhao

**Affiliations:** 1 Shanghai Sixth People’s Hospital, Shanghai Jiao Tong University School of Medicine, Shanghai, China; 2 Shanghai Ninth People’s Hospital, Shanghai Jiao Tong University School of Medicine, Shanghai, China

**Keywords:** Giant Cell Tumor of bone, Aneurysmal bone Cyst, hand short bone, huge bone tumor

## Abstract

**Background:**

Aneurysmal bone cyst (ABC) secondary to Giant Cell Tumor of bone (GCT) is a rare lesion, of which the incidence is about 0.011 to 0.053 per 100,000 every year. There are only a few previous case reports, and most of them occur in the spine, long bones or flat bones.

**Case presentation:**

We report one case of a patient who complained of “progressive enlargement of the mass on right-hand fifth finger for 5 years with ulceration for 6 months”. After the imaging examination in our hospital, it was diagnosed as a “huge bone tumor on the proximal phalanx of the right-hand fifth finger”, then wide excision and amputation of the fifth finger were made. The pathological examination diagnosed the mass as aneurysmal bone cyst secondary to giant cell tumor, 13 × 8 × 6 cm^3^, with no local infiltration observed. No recurrence and metastasis occurred 18 months after the operation, and the patient recovered well.

**Conclusion:**

In this report, we discuss the etiology, diagnosis, differentiation, and management of Aneurysmal bone Cyst secondary to Giant Cell Tumor of bone, and review previous case studies.

## Background

Giant cell tumor (GCT) of bone is a locally invasive tumor, mostly benign, but with a distant metastasis rate of 2% [1]. It is a common benign lesion in Asian, accounting for about 20% of all primary bone tumors. Most of GCTs arise in the epiphysis and are commonly found in the distal femur, proximal humerus, proximal femur, and distal tibia. Only about 0.5% occur in the hand [2], but the recurrence rate of them is higher than that in other parts [3]. Aneurysmal bone cysts (ABCs) account for about 5 to 6% of benign bone tumors [4] and mostly occur in young people before the age of 20. ABCs are considered primary lesions in approximately 70% of cases, with the remaining 30% arising secondary to different primary tumors. GCT is the most common primary lesion to secondary ABCs, accounting for 19 to 39% [5], and about 80% of GCT secondary to ABC occur in spine, long bones or flat bones [6]. According to the literature, there are few cases of GCT secondary to ABC occurring in the short bones of the hands or feet. This patient has a long course of the disease, the tumor is huge, and its clinical manifestation is not obvious. Additionally, the early stage is easily confused with other bone diseases such as tophus, so the article reviews and analyzes this case.

## Case presentation

**Fig. 1:**
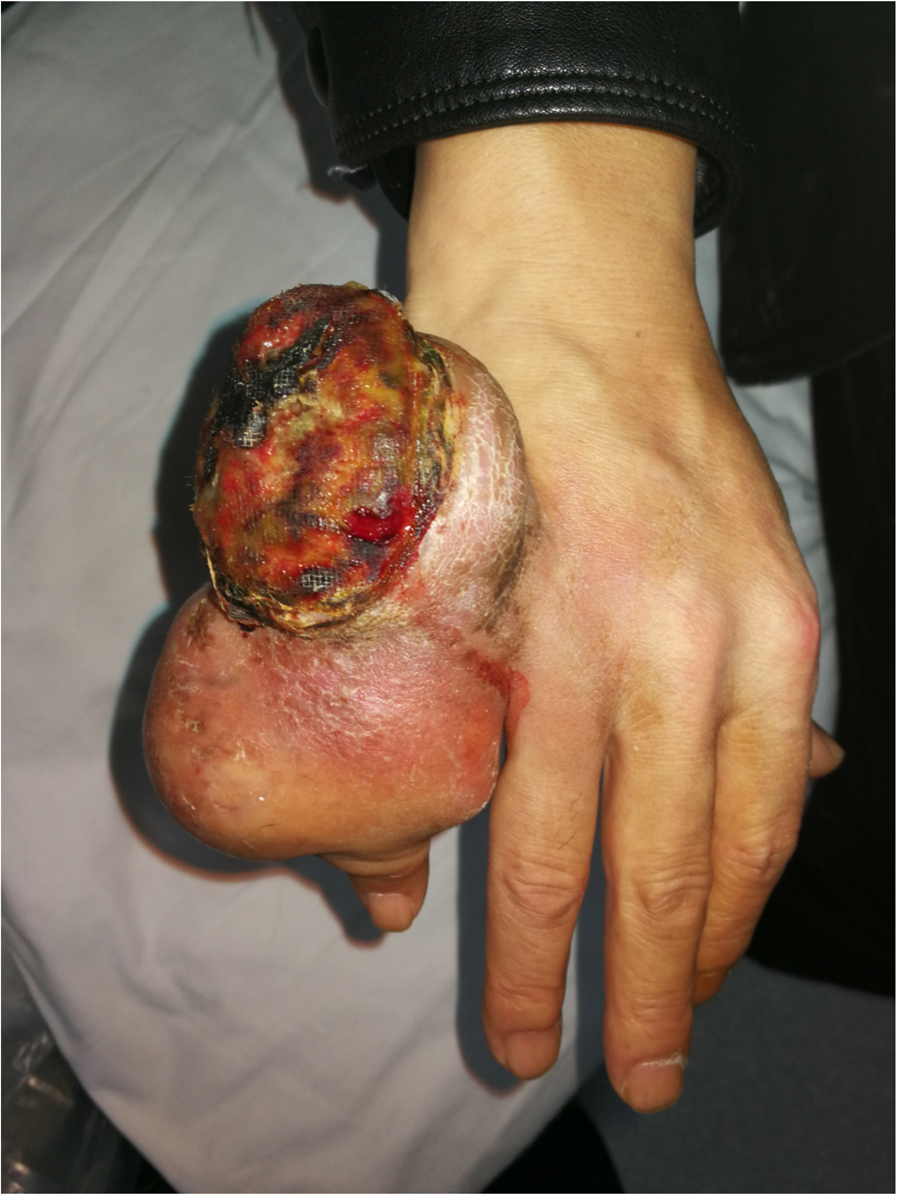
A huge mass can be seen at the proximal of right hand fifth finger, about 13 × 8 × 6 cm3, the surface collapsed, with a little bloody exudation

A 57-year-old man found a small mass on the proximal phalanx of the right fifth finger 5 years before. It was initially peanut-sized and slowly progressing without any discomfort like pain. About 1 year before, the mass increased rapidly and the patient went to another hospital after half a year. Because of the gout history of the patient, the doctor considered the mass tophus, but no obvious effect appeared after drug therapy. 2 months before, the mass has swollen to 13 × 8 × 6 cm^3^ (Fig. 1). The surface was partially broken, with no pain, no neuralgia, no sensory disorder, no headache or dizziness, no chest stuffy or shortness of breath, no nausea or vomiting, etc. It was initially diagnosed as a “huge bone tumor on the proximal phalanx of the right-hand fifth finger”. The patient has a history of smoking and drinking.

Upon physical examination, the mass on the ulnar of proximal interphalangeal joint of right-hand fifth fingers was about 13 × 8 × 6 cm^3^, tough touched, with slight tenderness, surface ulceration, a little bloody exudation, and no purulent secretions. The skin over the mass was warm, and the feeling and movement of the finger were normal. The axillary lymph nodes and supraclavicular lymph nodes were not swollen. The remaining bones of the limbs had no subcutaneous nodules. The liver and spleen were of normal sizes and there was no abnormality in the heart, lung, and brain.

**Fig. 2:**
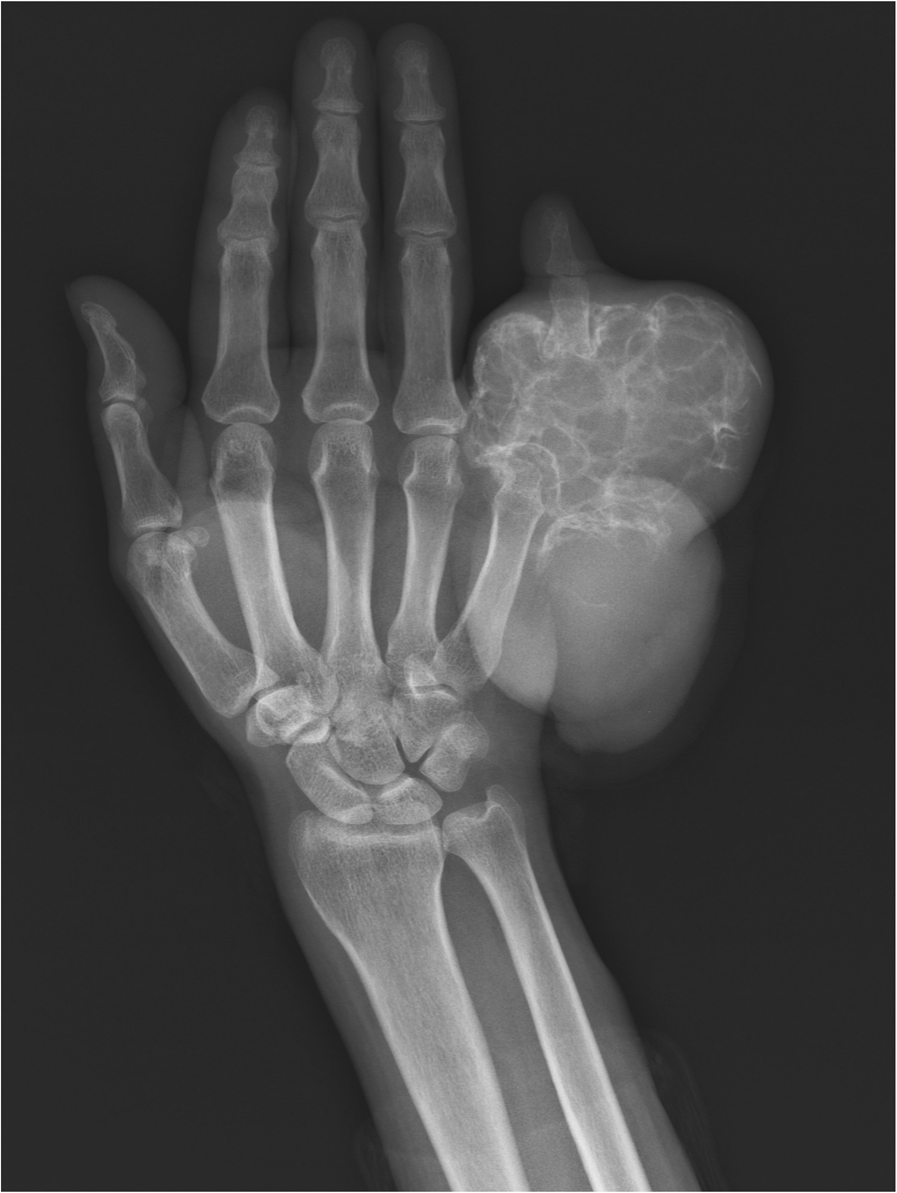
X-ray plain shows bone destruction, with swelling, bubble-like changes and local reticular shadow, accompanied by soft tissue swelling

X-ray plain (Fig. 2) of the right upper limb was performed. Right-hand fifth finger proximal phalanx bone destruction was seen, with swelling, bubble-like changes and local reticular shadow, accompanied by soft tissue swelling. Adjacent phalanx bone structure stayed clear.

**Fig. 3:**
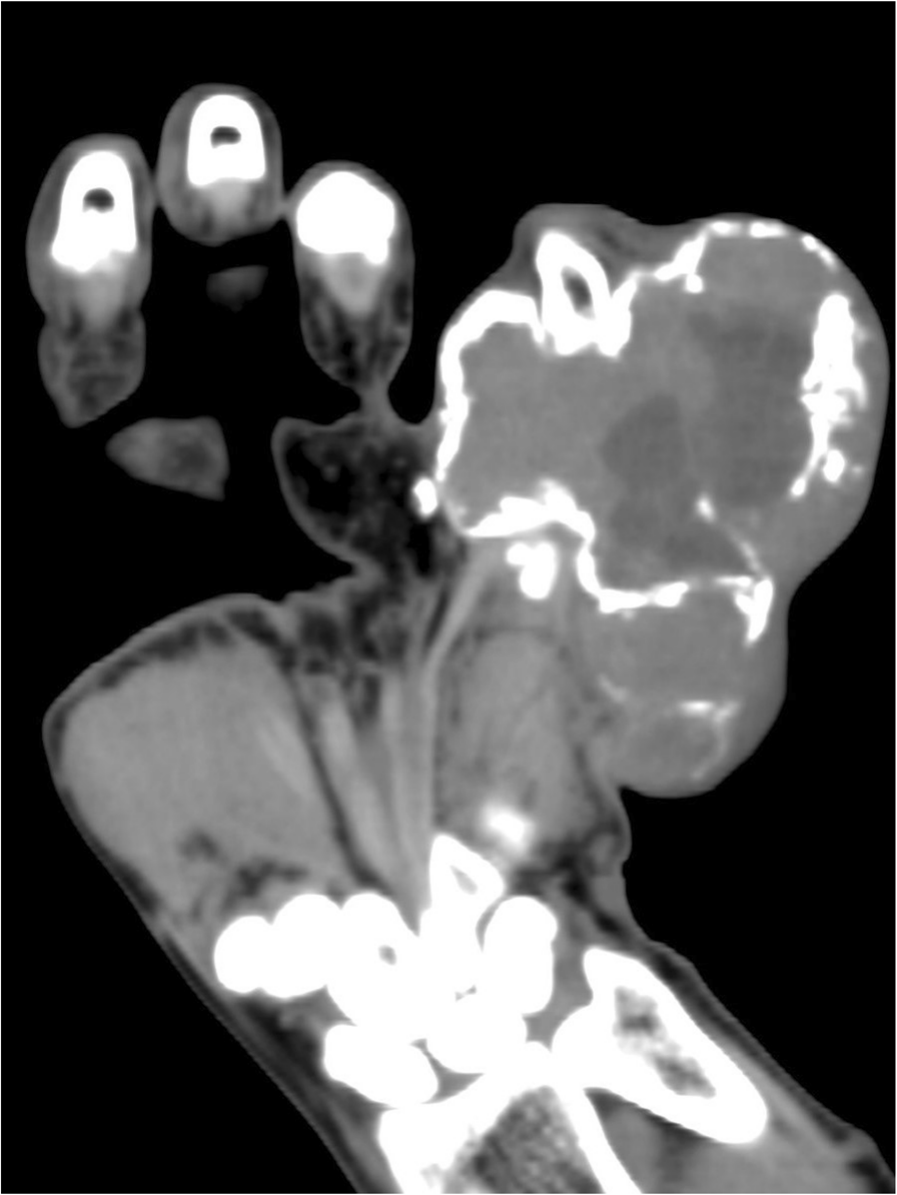
Coronal CT shows swelling changes, the bone shell is thin. The multilocular cystic division and multiple low-density shadows was seen

**Fig. 4:**
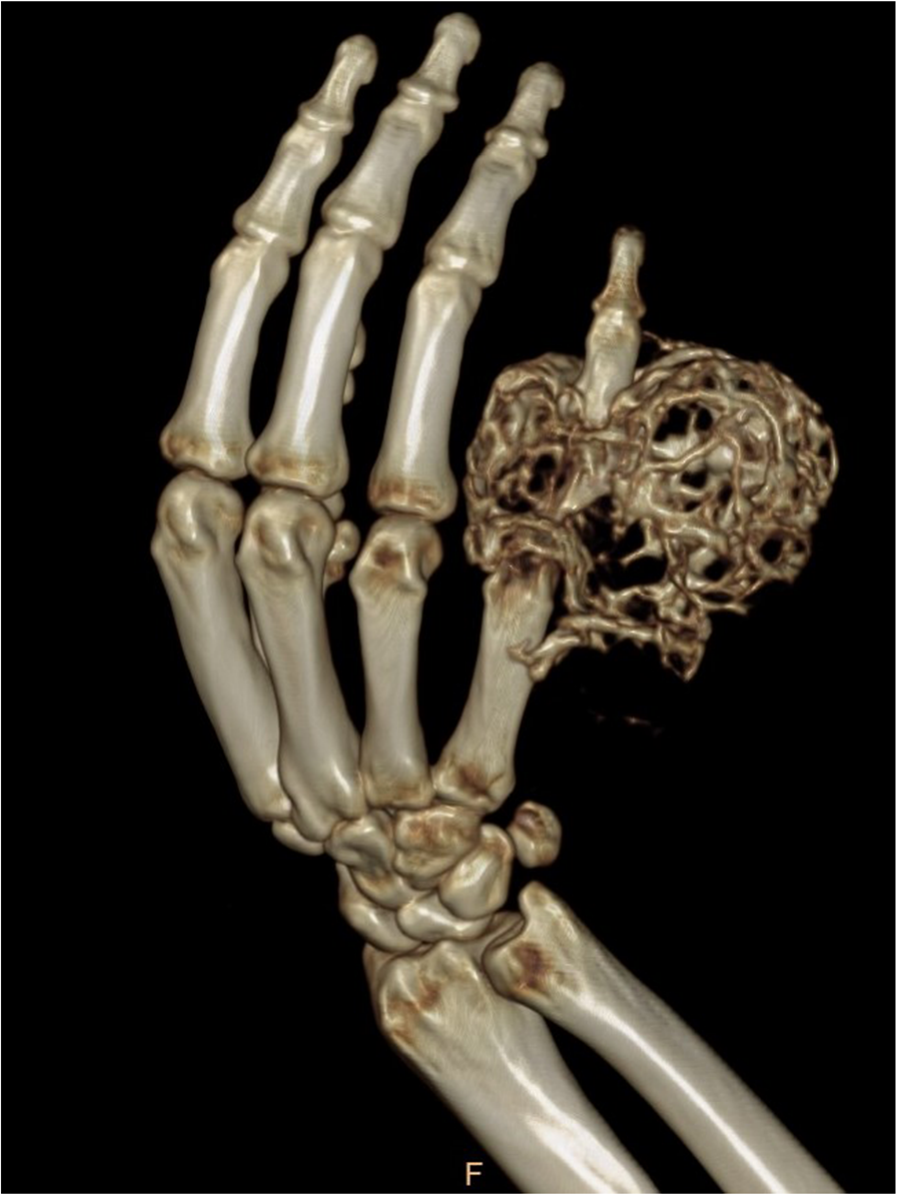
CT three-dimensional reconstruction showed soap bubble-like, swelling changes, osteolysis and bone mineralization existed simultaneously

Computed tomography (CT) three-dimensional imaging (Figs. 3 and 4) showed the mass on the right-hand fifth finger was about 8.6 × 6.3 × 5.8 cm3. The bone shell was thin. The multilocular cystic division and multiple low-density shadows were seen. Osteolysis and bone mineralization existed simultaneously. Part of the bone cortex was discontinuous, but the periosteal reaction was not obvious. A soft tissue mass was prominently deserved and the density of it was uneven, for the CT value was from 13 to 45 HU. The marginal part of mass bypassed the proximal interphalangeal joint and invaded the right middle fifth phalanx. It is a pity that MRI was not taken. X-ray of the chest revealed no pulmonary metastasis. Following laboratory tests, the blood uric acid was observed to be at 400umol/L and other test results were almost within the normal ranges.

**Fig. 5:**
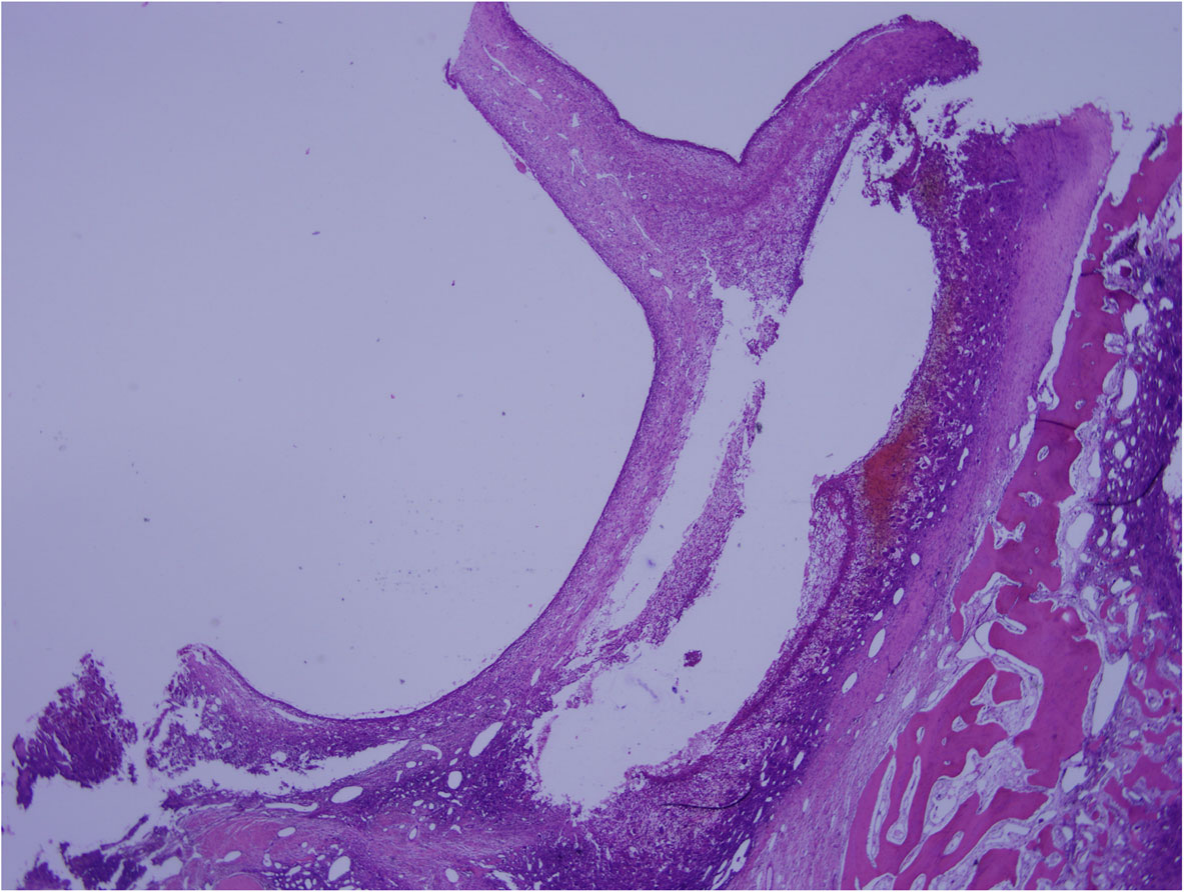
Pathology shows giant cell tumor of bone with aneurysmal bone cyst, the wall of the aneurysm can be seen (× 20)

**Fig. 6:**
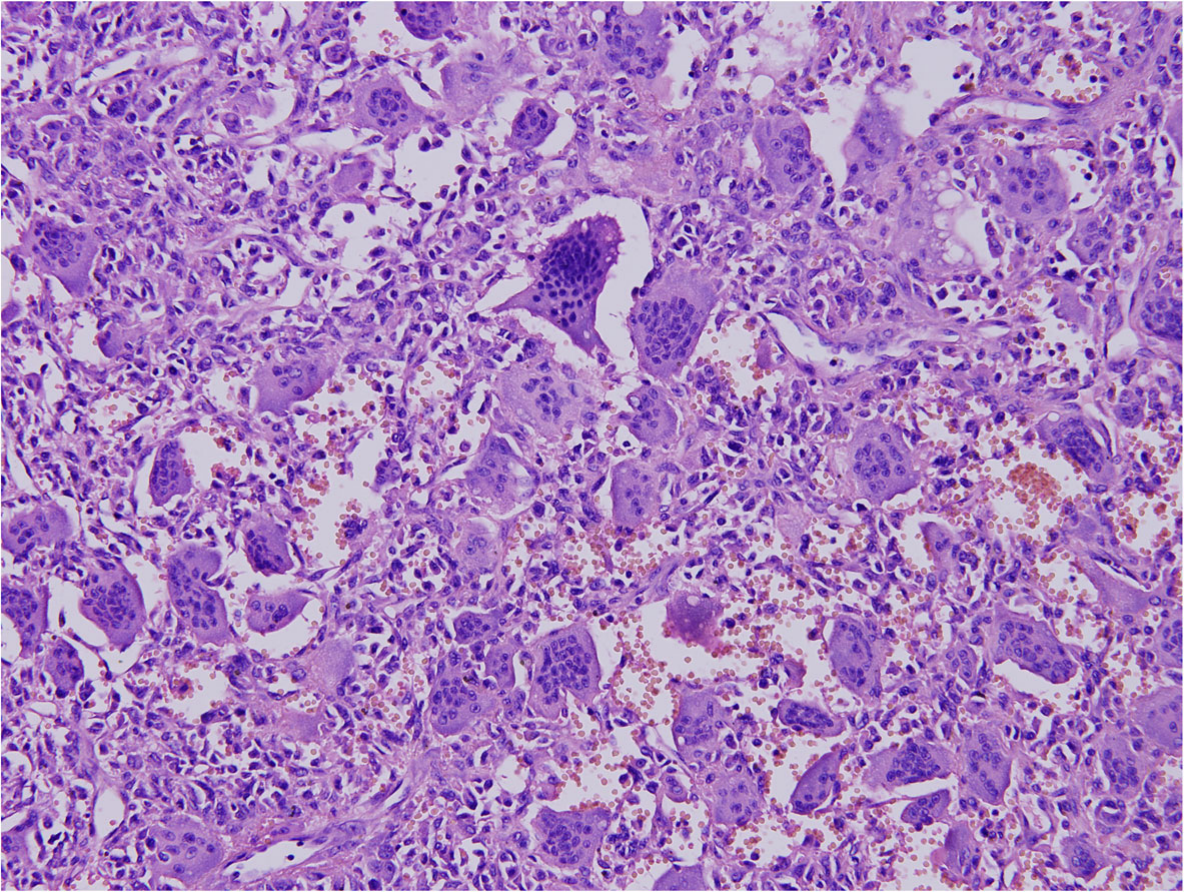
The giant cell tumor-like changes can be seen (× 200), and the arrows refer to the bone giant cells

The right-hand fifth finger interception combined with giant bone tumor resection was taken under the general anesthesia. Intraoperatively, the fifth metacarpal, phalanx, and tumor were completely removed. Histopathological findings revealed features of GCT with ABC (Figs. 5 and 6). As we can see, there are lots of mononuclear ovoid cells and multinucleated giant cells, and the big cell nuclei of them are mostly vacuolar. In another picture, the capsule wall of aneurysmal bone cyst and many small vessel walls can be seen, which is the feature of ABCs. Macroscopically, the resected tumor tissue was grey, red and white; no necrosis was observed. Microscopically, the resected margin was tumor-free. Immunohistochemistry results indicated that the tumor cells were partly positive for Vim, KP-1, PGM-1, and CD34, and negative for Des, MSA, P63, and CD45. The Ki67 was about 10%.

Following surgery, the patient’s vital signs were stable, and there was a small amount of exudation in the surgical incision. Due to the high skin tension of the local suture, the wound healing was a little poor, but the blood supply of fingertips was good. After several dressing changes the wound improved. No radiotherapy and chemotherapy were required, for the pathology suggested a benign change. Every one-month follow-up observation was required, clinical and radiological examinations, including palpation and plain radiography, were performed. No local recurrence or distant metastasis were identified 18 months following surgery. The patient is in a good position now.

## Discussion and Conclusions

Giant cell tumor of bone is a common local invasive tumor, mostly benign, accounting for 5% of all primary bone tumors in the Western population, and only 2–5% occurs in the phalanges [6]. Athanasian reported 13 cases of giant cell tumor on the phalanges, the clinical manifestation was mainly swelling (85%) and pain (62%), and some were found through physical examination (54%), fewer patients got pathological fractures (9%) [3]. Wold and Swee pointed out 13% of patients had multicentric giant-cell tumor [7]. Two of the 28 patients with giant cell tumor of the phalanx reported by Averill et al. had lung metastases [8]. Therefore, the CT of the chest and whole-body bone scans are considered as necessary examinations for the patient.

Histologically, giant cell tumor of bone is composed of mononuclear ovoid and spindle-shaped cells associated with multinucleated giant cells and macrophages [6]. The lesions were graded according to the appearance of the stroma and the giant-cells. Averill [8] et al. reported 28 cases, of which 12 were grade I, 3 were mixed of grade I and II, 4 were grade II and 9 were grade III. Among the common treatments, the recurrence rate of curettage alone or curettage with bone grafting was 79%, and the recurrence rate of local resection and bone grafting, ray resection or amputation was 36%. Radiation resection and extensive resection are deemed to be the most effective methods [8].

Aneurysmal bone cyst (ABC) was first described by Jaffe and Lichtenstein [9] in 1942 and was named for its pathological manifestations. Histologically, it is characterized by a cavernous vascular tumor ranging from a few millimeters to 1 to 2 cm in diameter, with intralesional communicating cavitations without blood clots. Typically, microscopic analysis of ABC reveals hemorrhagic tissue with cavitary spaces separated by fibrous septa composed of spindle cells, inflammatory cells, and a smaller percentage of giant cells [10]. Aneurysmal bone cyst is a benign, osteolytic, expansive, hemorrhagic and mostly solitary lesion, accounting for about 5 to 6% of benign bone tumors. Aneurysmal bone cysts can occur in bones throughout the body, especially in long bones (67%), spine (15%), and pelvis (9%). Frassica et al. reported 10 cases of ABC on phalanx, usually, patients felt pain (90%) and swelling (40%) and a few pathological fractures happened (10%) [11].

At present, the treatments of primary aneurysmal bone cysts mainly include nutritional vascular embolization, lesion scraping, surgical resection, and autologous bone transplantation. In the report of Frassica et al., the recurrence rate of lesion scraping and autologous bone graft is high, about 57.1%. 3 patients with complete bone resection and no recurrence happened [11].

Aneurysmal bone cyst secondary to giant cell tumor mostly occurs in patients of 20 to 40 years [12]. Recent studies suggest that secondary aneurysmal bone cysts may be related to factors such as hyperemia and dilatation of the vascular bed [13] or induced vascular bed caused by arteriovenous malformation of primary lesions [14]. Patients often present with intermittent pain, soft tissue mass, limited joint activity, etc. The incidence of pathological fracture is larger than that of patients with primary giant cell tumor of the bone [15].

Imaging examination is an important diagnostic tool for GCT and ABC. It is difficult for a simple X-ray examination to find two lesions, so CT and MRI are necessary supplements. CT typically revealed a characteristic soap-bubble appearance, and a balloon-like, multilocular lytic lesion. Pathologic fracture was a common finding. Magnetic resonance (MR) imaging revealed a mass with a fluid-fluid interface with hypointense signals on T1-weighted imaging (T1WI) and hyperintense signals on T2-weighted imaging (T2WI) with contrast enhancement of the septa. The presence of a double density fluid level within the lesion was often seen [12].

Histopathologically, ABC secondary to GCT shows two lesions at the same time microscopically, which is the basis for diagnosis. The treatment of ABC secondary to GCT is usually focused on the treatment of the bone giant cell tumor. The surgical method should be determined according to the degree of malignancy, location and peripheral invasion or not of bone giant cell tumor [16].

Identification with finger tophus: tophus is the white crystalline substance within the subcutaneous tissues or associated with joints and tendons, which can infiltrate the joint or tendon tissue and show sodium urate crystal under polarized light microscopy [17]. Typical sites for tophus deposition are well recognized including the olecranon bursa, the Achilles tendon, the first metatarsophalangeal joint, the ear and the finger pulps. The tophus is often multiple [18] and can cause joint damage, ankylosis, and others. Tophus is usually found in patients with a history of gout for more than 10 years without treatment [16]. On the X-ray plain, the tophus is often characterized by joint destruction, soft tissue swelling around the joint, and local uplift. In this case, the patient got hyperuricemia about 4 years ago. The location and clinical manifestations of the mass were similar with those of tophus. But such a huge tumor didn’t erode the joint, and the other limbs had no subcutaneous nodules, which can be the point distinguishing from tophus.

Differentiation from primary malignancy in giant cell tumor of bone (PMGCT): As is reported by Franco Bertoni [19], PMGCT is extremely unusual, there are only 5 PMGCTs in 924 patients diagnosed by GCT. Pain (4 cases) and swelling (2 cases) were the most common symptoms of the malignancies. All PMGCTs appeared on plain films as osteolytic lesions with well-circumscribed margins in the epiphyses of long bones. An area of less distinct margins was present in two cases, and cortical breakthrough was observed in four cases. A soft tissue mass was seen on plain films in two of the five cases. In this case, the mass grows rapidly in the last year, and the clinical manifestations are similar with those of osteosarcoma. The density of lesion on CT is uneven, bone destruction and bone regeneration exist at the same time, so the mass may be diagnosed as the malignant bone tumor. However, there is no obvious periosteal reaction on the X-ray plain. The possibility of malignant transformation may be low, and the diagnosis can be confirmed by pathological examination.

In summary, medical history and imaging examination are the main methods to identify the ABC secondary to GCT. The pathological examination is the gold standard. The surgical method should be determined according to the state of bone giant cell tumor. This paper reports a rare case of ABC secondary to GCT on the phalanx, hoping to provide experience for early diagnosis and treatment.

## Data Availability

The data that support the findings of this study are available from Shanghai Ninth People’s Hospital, but restrictions apply to the availability of these data, which were used under license for the current study, and so are not publicly available. Data are however available from the authors upon reasonable request and with permission of Shanghai Ninth People’s Hospital. We make sure identifying/confidential patient data should not be shared.

## Abbreviations

ABC: Aneurysmal bone Cyst
GCT: Giant Cell Tumor;
CT: Computed tomography
MRI: Magnatic Resonance Imaging
US: Ultrasonography
DECT: Dual-energy computed tomography
PMGCT: Primary malignancy in giant cell tumor of bone
VIM: Vimentin
PGM-1: Phosphoglucomutase-1
CD34: Cell differentiation-34
MSA: Multiplication-stimulating factor
